# Early head injury and attention deficit hyperactivity disorder: retrospective cohort study

**DOI:** 10.1136/bmj.a1984

**Published:** 2008-11-06

**Authors:** Heather T Keenan, Gillian C Hall, Stephen W Marshall

**Affiliations:** 1 Department of Pediatrics, University of Utah, Salt Lake City, UT 84158, USA; 2 Grimsdyke House, Ravenscroft Park, Barnet, Hertfordshire EN5 4ND; 3 Department of Epidemiology, School of Public Health, University of North Carolina at Chapel Hill, Chapel Hill, NC 27699, USA

## Abstract

**Objective** To explore the hypothesis that medically attended head injury in young children may be causal in the later development of attention deficit hyperactivity disorder.

**Design** Retrospective cohort study.

**Setting** Health improvement network database (1988-2003), a longitudinal UK general practice dataset.

**Participants** All children registered in the database from birth until their 10th birthday.

**Main outcome measures** Risk of a child with a head injury before age 2 developing attention deficit hyperactivity disorder before age 10 compared with children with a burn injury before age 2 and children with neither a burn nor a head injury.

**Results** Of the 62 088 children who comprised the cohort, 2782 (4.5%) had a head injury and 1116 (1.8%) had a burn injury. The risk of diagnosis of attention deficit hyperactivity disorder before 10 years of age after adjustment for sex, prematurity, socioeconomic status, and practice identification number was similar in the head injury (relative risk 1.9, 95% confidence interval 1.5 to 2.5) and burn injury groups (1.7, 1.2 to 2.5) compared with all other children.

**Discussion** Medically attended head injury before 2 years of age does not seem to be causal in the development of attention deficit hyperactivity disorder. Medically attended injury before 2 years of age may be a marker for subsequent diagnosis of attention deficit hyperactivity disorder.

## Introduction

An established association exists between attention deficit hyperactivity disorder (ADHD) and head injury, but the direction of the relation has been difficult to elucidate. Studies have suggested both that injury is more common in children with ADHD and that moderate to severe traumatic brain injury in school age children results in the development of ADHD (secondary ADHD).^1^ Further complicating the association is evidence that children in whom ADHD was diagnosed before they sustained a head injury may have more severe and persistent ADHD symptoms than those who were behaviourally asymptomatic before a head injury.^1^ ^2^ Mild brain injury in children has been associated with risk for psychiatric illness, especially hyperactivity,^3^ but this remains controversial.^4^ Because the behavioural difficulties associated with ADHD that follow a head injury may prevent children from achieving scholastically and socially even when their cognition is preserved, understanding the nature of the association is important.

We hypothesised that children with a medically attended head injury before 2 years of age would be more likely to be subsequently diagnosed as having ADHD than children who did not have a medically attended head injury before age 2. To investigate this, we selected two comparison groups: children who had a medically attended burn or scald injury without a head injury before their second birthday and children with neither a head injury nor a burn injury before age 2. If head injury is causal in the development of ADHD, we would expect the risk of ADHD to be higher in the head injury group, relative to both the burn injury group and the comparison group. We used two comparison groups (burns and all others) because we were concerned that factors associated with general injury and ADHD could confound our examination of the head injury-ADHD association.

## Methods

Data came from the health improvement network,^5^ a longitudinal dataset from primary care practices in the United Kingdom. The health improvement network contains information collected in computerised primary care records from 308 practices throughout the UK. Details of demographics, primary care diagnoses, and treatments prescribed are prospectively recorded in individual patient’s records. Details of referrals, secondary care diagnoses, and deaths are also captured because of the structure of the UK healthcare system. Within this system, the population are registered with one general practitioner and remain on that general practitioner’s list while being treated in secondary care. Medical events are automatically coded at entry by use of the READ coding system. Each primary care practice participating in the database has a unique practice identification number recorded in the dataset. The quality of the database is monitored, and medical diagnoses in the database have high validity.^6^

The study cohort included children who had a date of birth recorded in the health improvement network database since the inception of computerised data collection and continuous enrolment until they received a diagnosis of ADHD or until their 10th birthday. The study period was 1988 to 2003. We excluded children with a diagnosis of ADHD before their second birthday. We considered children to have ADHD if the READ code for ADHD, overactive child, attention deficit disorder, hyperkinetic disorder, or hyperkinetic conduct disorder was coded any time after the child turned 2 years of age. We extracted data from the records of children for as long as they remained registered in the database; however, we noted injuries, diagnoses, and prescriptions as occurring before or after the child’s 10th birthday.

We divided the study cohort into three groups. We defined the first group, children with early head injury, as children whose record contained any READ code for head injury except for “minor head injury” and “nursing advice for head injury” before the child’s second birthday. READ codes are sufficiently specific to differentiate abrasions and lacerations to the head and face from head injury itself. We extracted information on the source of the referral for patients with early head injury and dichotomised the data according to whether or not the child was referred by a physician to a higher level of care or admitted to hospital. The second group, children with early burn or scald injury, comprised all children who had a READ code for burn or scald recorded before their second birthday. Children from the cohort without head injury or burn injury before age 2 years made up the third group (comparison group).

Data extracted for all children included practice identification number, date of registration in the database, duration of registration, date of birth, sex, codes and dates of head injuries (up to three injuries after 2 years of age) and burn or scald injury, and any code indicating prematurity (<37 weeks gestational age) or child abuse. A head injury had to occur at least two months after a previous one to be considered a new injury, to ensure that the visit was not a follow-up visit. For each child, we extracted the date of the first diagnostic code for ADHD and the date of the first prescription for drugs commonly used for ADHD, including methylphenidate, dexamfetamine, and atomoxetine. We used the Townsend deprivation index, on the basis of patients’ home postcodes, to measure socioeconomic status. The Townsend index ranks levels of deprivation throughout the UK by fifths (1=least deprived; 5=most deprived).^7^ ^8^

### Data analysis

We used frequencies and percentages for categorical variables and medians with interquartile ranges for non-normally distributed continuous variables. We used the Wilcoxon-Mann-Whitney and the Kruskal-Wallis tests to compare continuous variables with skewed distributions. We considered P<0.05 to be statistically significant. We calculated the relative risk, with 95% confidence intervals, of developing ADHD for each injury group compared with the comparison group. We used χ^2^ tests to explore associations between predictor variables and the outcome. We did multivariate modelling, using the generalised estimating equation method to account for clustering by practice, to examine the association of injury type with the odds of developing ADHD after adjustment for Townsend index, sex, and prematurity. We used Poisson regression to calculate the unadjusted rate of diagnosis of ADHD.

## Results

### Study population

A total of 62 088 children in the health improvement network database met the study criteria (table 1). Of these, 2782 (4.5%) had a medically attended head injury before 2 years of age (head injury group) and 1116 (1.8%) had a medically attended burn or scald injury before age 2 (burn group). Study follow-up time was similar for both injured groups and the comparison group (P=0.08). Children with head injuries were slightly younger when injured (median 13.9 (interquartile range 9.0-18.4) months) compared with the burn injury group (14.8 (11.0-18.8) months) (P<0.001). Injured children were more likely to be male than non-injured children (P<0.001). The Townsend index showed greater deprivation among injured children than in the comparison group (P<0.001). The frequency of diagnosis of child abuse was low in all three groups (<0.1% in each group). A higher percentage of children who had had a head injury or burn injury before age 2 had medically attended head injuries after age 2 (P<0.001).

**Table 1 tbl1:** Characteristics of children aged under 2 years with head injury, those with burns and scalds, and comparison group. Values are numbers (percentages) unless stated otherwise

Characteristic	Head injury (n=2782)	Burns (n=1116)	Comparison (n=58 190)
Median (interquartile range) follow-up time (years)	12.4 (11.1-13.9)	12.5 (11.1-13.9)	12.5 (11.1-14.0)
Median (interquartile range) age at index injury (months)	13.9 (9.0-18.4)	14.8 (11.0-18.8)	NA
Males	1520 (54.6)	686 (61.5)	29 718 (51.1)
Premature	58 (2.1)	15 (1.3)	1 117 (1.9)
Townsend index*:	(n=2629)	(n=1052)	(n=55 786)
1 (least deprived)	665 (25.3)	274 (26.1)	15 431 (27.7)
2	502 (19.1)	188 (17.9)	11 547 (20.7)
3	537 (20.4)	203 (19.3)	11 209 (20.1)
4	443 (16.9)	189 (18.0)	9 252 (16.6)
5 (most deprived)	421 (16.0)	178 (16.9)	7 017 (12.6)
Missing	61 (2.3)	20 (1.8)	1 330 (2.4)
ADHD diagnosis:			
<10 years old	81 (2.9)	32 (2.9)	821 (1.4)
Ever	88 (3.2)	38 (3.4)	967 (1.7)
ADHD prescription:			
<10 years old	33 (1.2)	15 (1.3)	377 (0.7)
Ever	39 (1.4)	21 (1.9)	512 (0.9)
No of head injuries after age 2:			
1	418 (15.0)	123 (11.0)	4 502 (7.7)
2	79 (2.8)	21 (1.9)	558 (1.0)
3	12 (0.4)	4 (0.4)	74 (0.1)

ADHD=attention deficit hyperactivity disorder; NA=not applicable.

*Townsend index not calculated when denominator for any variable in index is less than 20.

### Diagnosis of ADHD

A diagnosis of ADHD after the second birthday and before the 10th birthday occurred in 1.5% (n=934) of the entire study cohort. The incidence of diagnosis of ADHD for the entire cohort was 1.2 (95% confidence interval 1.1 to 1.3) per 1000 person years before age 10 and 1.4 (1.3 to 1.5) per 1000 person years over the entire study period. Most children (769; 70.4%) with a diagnosis of ADHD had a diagnosis of primary ADHD; 284 (26.0%) had a diagnosis of hyperkinetic disorder, and 37 (3.4%) had a diagnosis of overactivity; only two (0.2%) children were diagnosed as having hyperkinetic conduct disorder. Compared with the comparison group, children in the two injury groups had a similarly increased likelihood of being diagnosed as having ADHD after the age of 2: relative risk 2.0 (95% confidence interval 1.6 to 2.5) for head injuries; 2.3 (1.6 to 3.1) for burn injuries. These two groups accounted for 6.3% (n=3898) of the study cohort and 11.3% (126) of the children in the cohort who went on to be diagnosed as having ADHD.

Treatment for ADHD was prescribed to 45.5% (425) of the entire cohort diagnosed as having ADHD before 10 years of age. Children with a history of burn or head injury were not more likely than their uninjured comparison counterparts to receive treatment once diagnosed. Most children (545; 95.3%) were treated with methylphenidate; relatively few were treated with atomoxetine (17; 3.0%) or dexamfetamine (10; 1.8%).

Children with head injury before age 2 were diagnosed as having ADHD at a slightly younger median age (6.2 (interquartile range 4.0-8.0) years) than children with burn injury (6.8 (4.8-9.0) years) or the comparison group (6.9 (4.6-8.8) years); however, this difference did not reach statistical significance (P=0.07). Male sex (relative risk 4.8, 4.0 to 5.7), a history of prematurity (1.6, 1.1 to 2.4), and a more deprived Townsend index (Cochran-Armitage test for trend, P<0.001) were all positively associated with diagnosis of ADHD.

The risk of diagnosis of ADHD before age 10 remained higher in both the head injury group (relative risk 1.9, 1.5 to 2.5) and the burn injury group (1.7, 1.2 to 2.5) compared with the comparison group, even after adjustment for sex, prematurity, Townsend index, and practice identification number (table 2). In addition, the risk of diagnosis of ADHD increased among children who had a head injury after age 2 in all three groups in the cohort (table 3).

**Table 2 tbl2:** Unadjusted and adjusted associations of early injury with diagnosis of attention deficit hyperactivity disorder (ADHD)

	Relative risk (95% CI) of ADHD diagnosis before age 10	Relative risk (95% CI) of ADHD diagnosis ever	Comparison
**Comparison group**			
Unadjusted	1.0 (referent)	1.0 (referent)	Referent
Adjusted*	1.0 (referent)	1.0 (referent)	Referent
**Head injury group**			
Unadjusted	2.0 (1.6 to 2.5)	1.9 (1.5 to 2.4)	Referent
Adjusted*	1.9 (1.5 to 2.5)	1.8 (1.4 to 2.2)	Referent
**Burn injury group**			
Unadjusted	2.3 (1.6 to 3.1)	2.0 (1.5 to 2.8)	Referent
Adjusted*	1.7 (1.2 to 2.5)	1.8 (1.3 to 2.5)	Referent

*Adjusted for sex, prematurity, Townsend index, and practice identification number.

**Table 3 tbl3:** Unadjusted association of number of head injuries with diagnosis of attention deficit hyperactivity disorder (ADHD)

No of head injuries before age 2	Relative risk (95% CI) of ADHD diagnosis before age 10
Comparison group:	
0	Referent
≥1	1.8 (1.5 to 2.1)
Head injury group:	
0	1.8 (1.4 to 2.4)
≥1	3.4 (2.3 to 5.0)
Burn injury group:	
0	1.9 (1.3 to 2.7)
≥1	2.9 (1.5 to 5.5)

## Discussion

This study found that children with a head injury before age 2 were twice as likely to be diagnosed as having ADHD as a population based comparison group but not more likely to be diagnosed as having ADHD than another medically attended injury group (burn injury). Thus, contrary to our hypothesis, the head injury itself does not seem to be causal in the development of ADHD. Rather, some other factor seems to be associated generally with early injury and the development of ADHD. These results indicate that medically attended injury before age 2 may be an early marker for behavioural traits that lead to diagnosis of ADHD.

We find it plausible that children who go on to develop clinical ADHD exhibit more risk taking behaviours as young children, and are therefore more likely to be injured before age 2. Consistent with this, children with a head injury or a burn injury before age 2 were also more likely to have medically attended head injuries after age 2 than were comparison children, suggesting that early injury is a marker of behavioural traits associated with ADHD.

Several studies have documented that children who have been diagnosed as having ADHD are more likely to be injured, possibly secondarily to poor impulse control and increased risk taking behaviour.^9^ ^10^ These studies identified children with ADHD and later injury. Our study identified early injury and then looked for the subsequent diagnosis of ADHD. It suggests that early head injury or burn injury is a marker for subsequent diagnosis of ADHD as well as future head injury. This is important, as children with ADHD before a head injury tend to fare more poorly in both cognition and adaptive behaviour after serious traumatic brain injury than do children without pre-injury behavioural concerns.^2^ ^11^ Additionally, children with ADHD may be more vulnerable to post-concussive syndrome after a mild traumatic brain injury.^12^ This would place this group of children at high risk for poor outcomes from subsequent head injuries.

### Strengths and limitations

This study is both limited and strengthened by the use of a large database. A limitation imposed by the dataset is the lack of detail about the seriousness of the injury event. The health improvement network database codes whether the child was admitted to hospital or seen in the emergency department for the injury by using source codes; however, this field was not coded for 50% of the patients. Whether an empty source field indicates missing data or that the patient was not referred to the general practitioner by another caregiver is not known. Severity of injury was unavailable from the diagnostic codes, as general practitioners tend to use one code indicating head injury regardless of severity (that is, the same code is used for an injury with a source code indicating hospital admission or outpatient referral). Secondary ADHD has been associated more strongly with severe and moderate traumatic brain injury than with mild injury.^13^ Thus, inclusion of some children with moderate to severe injury in this dataset would have tended to bias the results towards a higher risk of ADHD in the children with head injuries than in the controls with burns, which we did not find. Additionally, whether each diagnosis of ADHD met the *Diagnostic and Statistical Manual of Mental Disorders*, third edition, criteria for ADHD is not known, as children may not have received formal psychiatric evaluation. This is true for all children in the dataset, however. The overall prevalence of ADHD in this dataset is low compared with the prevalence estimates of ADHD in US children (3-7%) and similar to that reported in British Columbia (1.6%),^9^ ^14^ so the condition was probably not overdiagnosed.

Strengths of these data include the ability to follow children over time, to use a second injured control group, and to adjust for factors commonly related to both injury and ADHD. This allowed us to make reasonable estimates of the effect of early injury itself.

### Conclusions

These results show that medically attended head injury before age 2 does not seem to be causal in the development of ADHD, but rather any medically attended injury before age 2 may be a marker for subsequent diagnosis of ADHD. This study, one of the first to examine children under 2 years of age, should be replicated in a prospective cohort study that would allow for a finer definition of severity of injury and ADHD subtypes. The results emphasise the importance of obtaining pre-injury developmental status when examining the relation of head injury to neurodevelopmental consequences.

**What is already known on this topic**

Mild traumatic brain injury is thought to be associated with behavioural changes in children
Disentangling pre-existing behaviour problems from the sequelae of injury has been difficult

**What this study adds**

Medically attended head injury before age 2 does not seem to be causal in the development of attention deficit hyperactivity disorder
Any medically attended injury before age 2 may be a marker for subsequent diagnosis of attention deficit hyperactivity disorder

## Notes

We thank Mary Thompson and the team from EPIC for providing the health improvement network data and the general practitioners for their continued support of the dataset.

Ethical approval: Oxfordshire Multicentre Research Ethics Committee (ID# 06/Q1605/32) and the Institutional Review Board of the University of Utah gave approval for use of the health improvement network data.

Provenance and peer review: Not commissioned; externally peer reviewed.

## Notes

**Cite this as: ***BMJ* 2008;337:a1984
